# Silencing of CCR4-NOT complex subunits affects heart structure and function

**DOI:** 10.1242/dmm.044727

**Published:** 2020-07-20

**Authors:** Lisa Elmén, Claudia B. Volpato, Anaïs Kervadec, Santiago Pineda, Sreehari Kalvakuri, Nakissa N. Alayari, Luisa Foco, Peter P. Pramstaller, Karen Ocorr, Alessandra Rossini, Anthony Cammarato, Alexandre R. Colas, Andrew A. Hicks, Rolf Bodmer

**Affiliations:** 1 Development Aging and Regeneration Program, Sanford Burnham Prebys Medical Discovery Institute, 10901 N Torrey Pines Rd, La Jolla, CA 92037, USA; 2 Institute for Biomedicine, Eurac Research, Affiliated Institute of the University of Lübeck, Via Galvani 31, 39100 Bolzano, Italy; 3 Johns Hopkins University, Division of Cardiology, 720 Rutland Ave., Baltimore, MD 21205, USA

**Keywords:** CNOT1, GWAS, Arrhythmia, Long-QT syndrome, *Drosophila* heart, hiPSC, Cardiomyocytes

## Abstract

The identification of genetic variants that predispose individuals to cardiovascular disease and a better understanding of their targets would be highly advantageous. Genome-wide association studies have identified variants that associate with QT-interval length (a measure of myocardial repolarization). Three of the strongest associating variants (single-nucleotide polymorphisms) are located in the putative promotor region of *CNOT1*, a gene encoding the central CNOT1 subunit of CCR4-NOT: a multifunctional, conserved complex regulating gene expression and mRNA stability and turnover. We isolated the minimum fragment of the *CNOT1* promoter containing all three variants from individuals homozygous for the QT risk alleles and demonstrated that the haplotype associating with longer QT interval caused reduced reporter expression in a cardiac cell line, suggesting that reduced *CNOT1* expression might contribute to abnormal QT intervals. Systematic siRNA-mediated knockdown of CCR4-NOT components in human induced pluripotent stem cell-derived cardiomyocytes (hiPSC-CMs) revealed that silencing *CNOT1* and other CCR4-NOT genes reduced their proliferative capacity. Silencing *CNOT7* also shortened action potential duration. Furthermore, the cardiac-specific knockdown of *Drosophila* orthologs of CCR4-NOT genes *in vivo* (*CNOT1/Not1* and *CNOT7/8/Pop2*) was either lethal or resulted in dilated cardiomyopathy, reduced contractility or a propensity for arrhythmia. Silencing *CNOT2/Not2*, *CNOT4/Not4* and *CNOT6/6L/twin* also affected cardiac chamber size and contractility. Developmental studies suggested that *CNOT1/Not1* and *CNOT7/8/Pop2* are required during cardiac remodeling from larval to adult stages. To summarize, we have demonstrated how disease-associated genes identified by GWAS can be investigated by combining human cardiomyocyte cell-based and whole-organism *in vivo* heart models. Our results also suggest a potential link of *CNOT1* and *CNOT7/8* to QT alterations and further establish a crucial role of the CCR4-NOT complex in heart development and function.

This article has an associated First Person interview with the first author of the paper.

## Abstract (precis)

**Summary:** Genome-wide association studies combined with *in vitro* human cardiac cell assays and a model organism suitable for heart studies *in vivo* connect CNOT1, CNOT7 and overall the CCR4-NOT complex to human heart disease and morbidity.

## INTRODUCTION

Despite medical advances over the past few decades, cardiovascular disease remains the most common cause of mortality worldwide [https://www.who.int/news-room/fact-sheets/detail/cardiovascular-diseases-(cvds)]. Understanding the mechanisms of heart morbidity is crucial for finding new therapies, and determining which genetic variants predispose individuals to heart disease is necessary to provide better preventative care. The challenge of connecting human genetic variants with disease can be met by combining genome-wide association studies (GWAS) with patient sequencing and validation using disease-in-a-dish and *in vivo* cardiac model systems. *Drosophila melanogaster* benefits from well-conserved genes and permits functional assessment of genes of interest, which when manipulated might not be well tolerated by the vertebrate heart.

The QT interval on an electrocardiogram is a measure that reflects myocardial repolarization. Short-QT syndrome (Rudic et al., 2014) and long-QT syndrome (Amin et al., 2013) are caused by different underlying mechanisms, but are both risk factors for atrial and ventricular arrhythmias and sudden cardiac death (Rudic et al., 2014; Amin et al., 2013; Vacanti et al., 2017). Genome-wide association in up to 100,000 individuals has successfully identified at least 35 common variant QT-interval loci that collectively explain ∼8-10% of QT variation (Arking et al., 2014). Some of the strongest QT-associating variants identified center around the *CNOT1* gene, which encodes the central scaffolding subunit CCR4-NOT transcription complex subunit 1 (CNOT1) of the CCR4-NOT complex. CCR4-NOT is conserved throughout the eukaryotic kingdom and is involved in the sequential processes of gene expression. Its activities can be divided into functional modules involved in transcription (Kruk et al., 2011) (CNOT2, CNOT3), mRNA degradation (Bhandari et al., 2014; Yi et al., 2018; Temme et al., 2010; Webster et al., 2018), deadenylation (*CNOT6/6L*, *CNOT7/8*) and protein quality control through ubiquitination (*CNOT4*) (Halter et al., 2014; Collart, 2016; Collart and Panasenko, 2017).

The CCR4-NOT complex has previously been implicated in heart disease; we have demonstrated that silencing of genes *Ubc4* and *Not3* cause cardiac dilation and dysfunction in *Drosophila* (Neely et al., 2010). In addition, *CNOT3* heterozygous knockout mouse hearts displayed reduced contractility and increased susceptibility to failure following aortic constriction (Neely et al., 2010). CNOT3 has also been found to interact with Atg7, which affects cardiomyocyte (CM) survival and QT intervals in mice (Yamaguchi et al., 2018). In the present study, we investigated the individual role of additional CCR4-NOT complex subunits, starting with variants in the *CNOT1* putative promoter region that positively associate with QT intervals, to determine whether and in what direction these variants functionally influence reporter gene expression. We further explored the effects of RNA interference (RNAi)-mediated knockdown of *CNOT1* and complex subunit genes *CNOT2*, *CNOT4*, *CNOT6/6L* and *CNOT7/8* in human induced pluripotent stem cell-derived cardiomyocytes (hiPSC-CMs) (Cunningham et al., 2017; Yu et al., 2018); the effects of knockdown on proliferation and electrophysiological properties – in particular, action potential duration (APD) (McKeithan et al., 2017) – were investigated. In addition, the effects of gene knockdown on cardiac structure and contractile function were studied *in vivo* using the *Drosophila* heart model (Ocorr et al., 2014). Overall, we find that silencing *CNOT1* and other CCR4-NOT components compromises cardiac development and function in the two model systems, suggesting an important role of this complex in cardiac health and disease.

## RESULTS

### Functional validation of *CNOT1* promoter polymorphisms

**Fig. 1.**
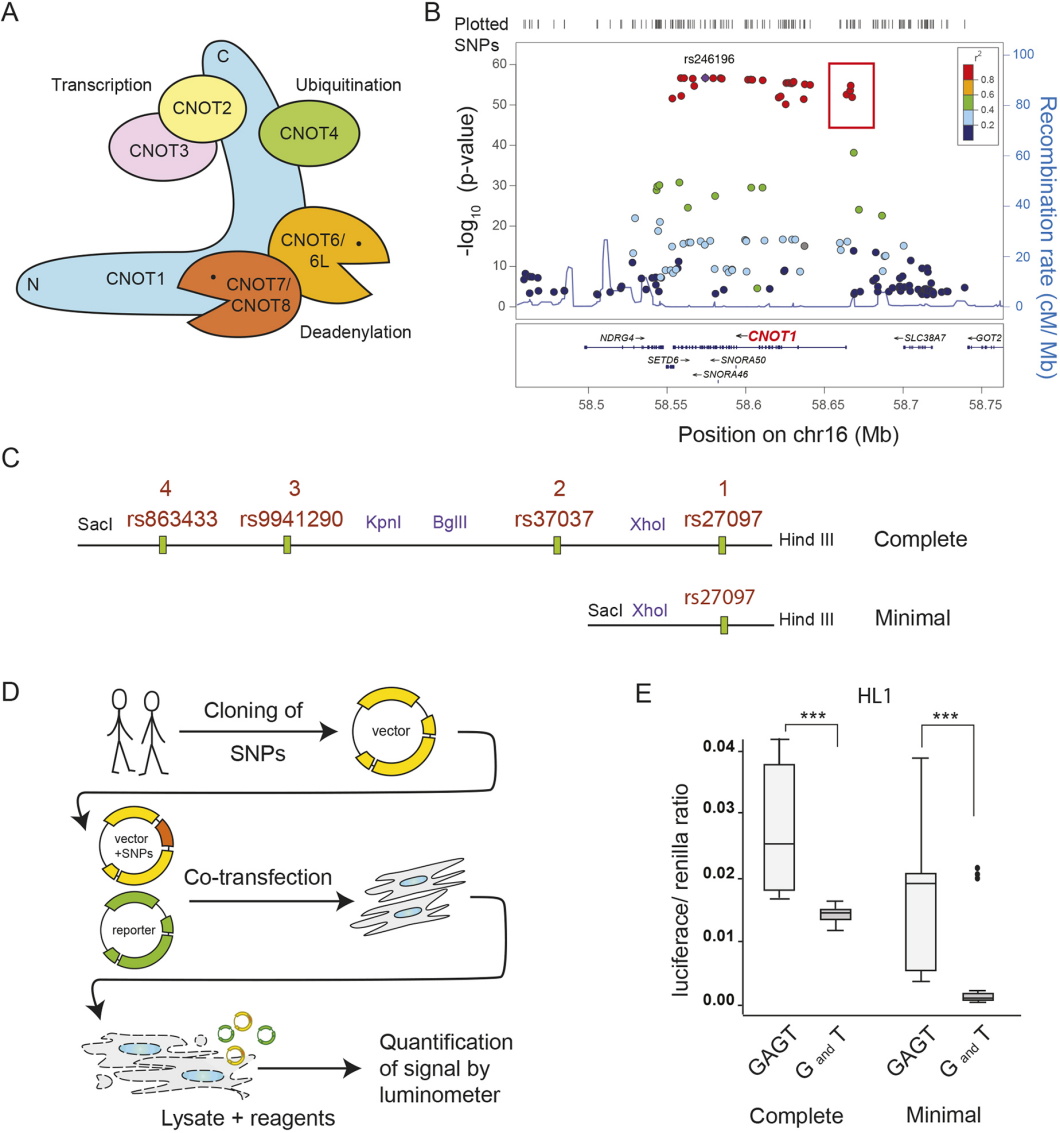
**The CCR4-NOT complex and functional validation of *CNOT1* promoter polymorphisms.** (A) Cartoon of the CCR4-NOT complex with the subunits investigated in this study. (B) GWAS identifies *CNOT1* SNPs associated with human QT syndrome. The four putative promoter SNPs are highlighted in the red box. Redrawn from fig. S2 in Arking et al. (2014). This image is not published under the terms of the CC-BY license of this article. For permission to reuse, please see Arking et al. (2014). (C) Constructs generated for the *CNOT1* complete promoter region and the minimal promoter region, cloned from two subjects carrying haplotype ‘GAGT’ and haplotype ‘TGAG’. Numbers above each SNP denote how close the variant is to the open reading frame (e.g. 1 is the closest). (D) Schematic of experimental procedure. (E) Box plots summarizing the intensity of the ratio between luciferase and renilla signal in HL1 cells. Boxes, interquartile range; central line, median; whiskers, upper and lower adjacent values as defined by Tukey (1977); dots, outside values. Two-sided *P*-values were computed using the Wilcoxon rank-sum test (***P≤0.001). For complete promoter, the number of independent experiments *n*=3; for each experiment, the number of independent biological replicates per haplotype *n*=3. Number of observations for each haplotype *n*=9, total *n*=18. For minimal promoter, the number of independent experiments *n*=5; for each experiment, the number of independent biological replicates per haplotype *n*=3, except one experiment (*n*=4). Number of observations for each haplotype *n*=16, total *n*=32.

Three of the strongest QT interval-associating variants [single-nucleotide polymorphisms (SNPs)], in strong linkage disequilibrium (LD) over *CNOT1*, are located in the putative promoter region of the gene (Fig. 1B; redrawn from fig. S2 in Arking et al., 2014). All GWAS-associated variants over the whole length of this gene are in strong LD with the putative promoter variants, and the Genotype-Tissue Expression (GTEx) data (https://doi.org/10.1089/bio.2015.0032) indicate strong tissue-specific expression quantitative trait loci (eQTL) variants over the whole gene; this observation led us to postulate that the promoter variants would demonstrate functionally different expression levels. Sequence analysis of a fragment spanning all three of these variants strongly associated with QT interval confirmed the presence of four SNPs (rs27097, rs37037, rs9941290 and rs863433). Three of these SNPs associate strongly with QT interval, whereas rs37037 (which is not in strong LD with the other three variants) is still associated but with a less significant *P*-value (Fig. S1C). We identified two human subjects homozygous for alleles at the four SNPs that fall within ∼3.2 kb of the 5′ region of the *CNOT1* coding sequence (Fig. 1C). The two haplotypes (one with risk alleles and one with alternate alleles) from the putative *CNOT1* promoter region were isolated and cloned in two forms into a plasmid vector (pGL4.1) to drive the firefly luciferase gene (Fig. 1D). The ‘minimal’ putative promoter region contained 657 bp of the region around just one SNP (rs27097), located closest to the start codon of the *CNOT1* gene. The larger 3172 bp fragment contained all of the strongest QT interval-associating variants, potentially capturing the ‘complete’ promoter region of *CNOT1* (with respect to the significantly associated variants in this region of the gene). Both constructs were sequenced to confirm that the four variant positions under study were the only ones differing between these natural promoter regions and that the alleles were homozygous. For both the minimal and complete constructs, one haplotype consisted of alleles that significantly associate with increases in QT interval length (‘TGAG’ haplotype), whereas the other haplotype consisted of the alternate alleles at these variants (‘GAGT’ haplotype) (see also Fig. S1C) (Pfeufer et al., 2009). The minimal promoter constructs consisted of alleles T and G, respectively, at rs27097 (Fig. 1C).

The complete and minimal promoter-luciferase constructs were transfected into HL-1 cells, a cardiac muscle cell line that contracts and retains phenotypic characteristics of adult CM, along with renilla luciferase in order to normalize signals. Both minimal and complete promoter constructs were able to drive increased luciferase expression in the HL-1 cells with significant differences between the two haplotypes. When considering the SNP closest to the gene alone (minimal construct), expression was effectively silenced in the HL-1 cell line for the haplotype that was associating with longer QT intervals (Fig. 1E). These differences were also observed in both HeLa and T293 cells (Fig. S1A,B). With the larger promoter fragment, expression was re-established in all lines, but was significantly reduced for the haplotype associated with a longer QT interval compared with the haplotype carrying the alternate alleles. From these experiments, we conclude that the variants that significantly associate with QT interval in human GWAS are indeed functionally able to alter expression of the *CNOT1* gene in cardiac tissue, with the most significant differences for the complete haplotype being seen in the cardiac cell line. The direction of the effect is such that reduced *CNOT1* possibly contributes to QT-interval prolongation.

### Genes encoding the CCR4-NOT complex regulate proliferation and APD in hiPSC-CMs

CCR4-NOT is a multisubunit complex with different functional modules (including, but not limited to, the subunits considered in this study) (Fig. 1A). It has been shown in HeLa and HEK293T cells that short interfering RNA (siRNA) depletion of *CNOT1* decreases the amount of CCR4-NOT subunits and reduces deadenylase activity of the complex; simultaneous siRNA silencing of the entire deadenylase module (*CNOT6/6L*, *CNOT7/8*) results in apoptosis similar to that of *CNOT1* silencing alone (Ito et al., 2011). Therefore, we decided to use hiPSC-CMs to investigate the effect of silencing *CNOT6/6L* and *CNOT7/8*, genes encoding enzymes that affect translation efficiency, by removing the mRNA poly(A) tail (Yi et al., 2018). This approach was also used to study the gene *CNOT4*/*Not4*, which encodes a RING E3-ligase important for assembly of the proteasome and proposed to be involved in co-translational quality control (Halter et al., 2014). We also chose to examine *CNOT2*, as it associates with *CNOT3*, for which we previously identified a role in cardiac function (Neely et al., 2010).

**Fig. 2.**
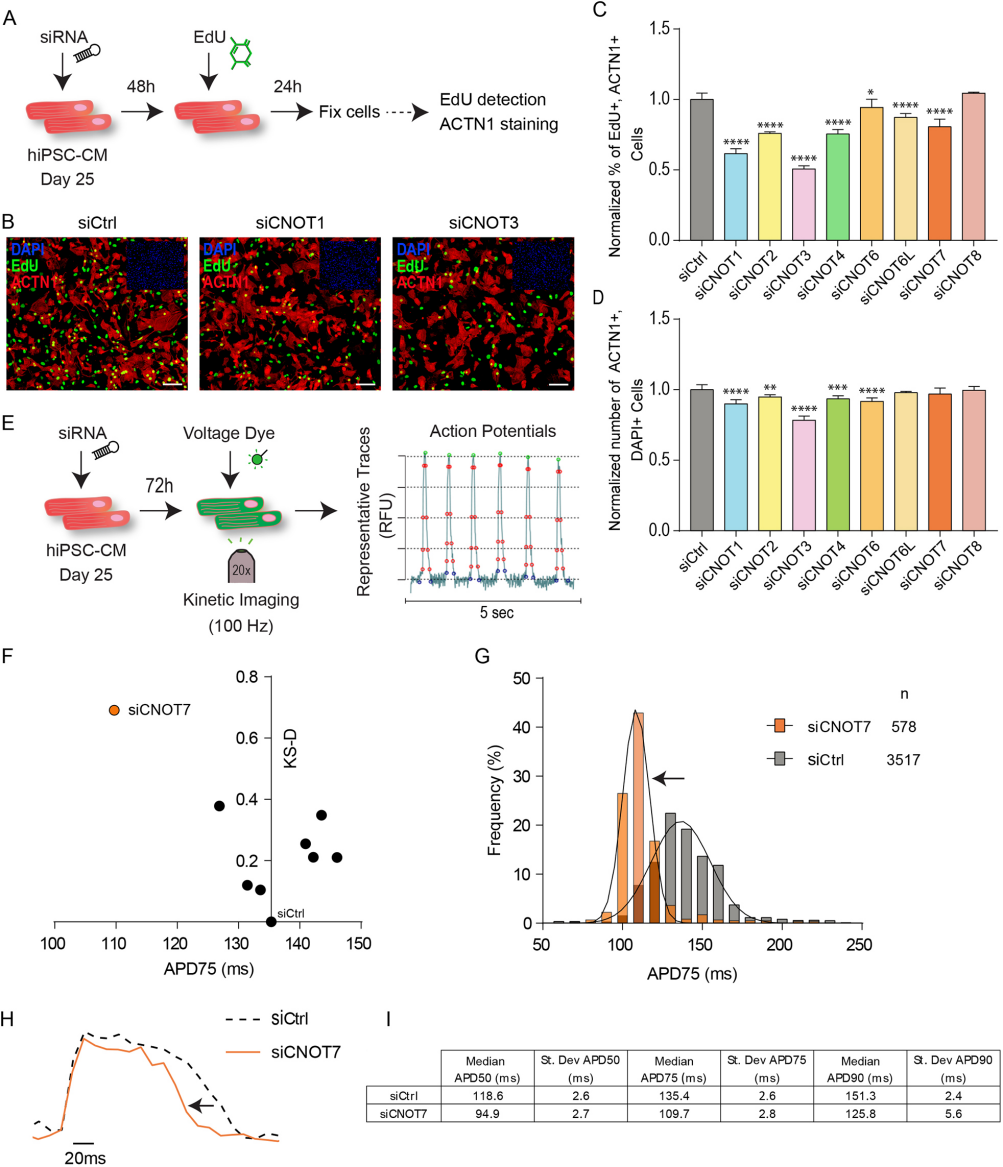
**Genes encoding the CCR4-NOT complex regulate proliferation and action potential in hiPSC-CMs.** (A) Schematic of the proliferation assay in hiPSC-CMs. (B) Representative immunofluorescence images for EdU and ACTN1 in control (siCtrl), siCNOT1 and siCNOT3 conditions. Scale bars: 25 µm. (C,D) Histograms showing the normalized percentage of EdU-positive hiPSC-CMs and normalized number of hiPSC-CMs. Student's *t*-test was used to calculate *P*-values. *P≤0.05, **P≤0.01, ***P≤0.001, ****P≤0.0001. (E) Schematic overview of the single-cell, high-throughput voltage assay. RFU, relative fluorescence units. (F) Two-dimensional graph for APD75 and Kolmogorov-Smirnov distance (KS-D) representing screen results for CCR4-NOT component knockdown. (G) Population distribution of APD75 measurements for siCNOT7- versus siCtrl-transfected hiPSC-CMs. (H) Median action potential traces for siCNOT7*-* and siCtrl-transfected hiPSC-CMs. (I) Table summarizing average and s.d. values for APD50, APD75 and APD90 for siCNOT7- and siCtrl-transfected hiPSC-CMs.

First, to evaluate a potential role of the CCR4-NOT complex in human cardiac physiology, we knocked down each of the CNOT genes and evaluated their effect on hiPSC-CM proliferation. Individual knockdown of *CNOT1*, *CNOT2*, *CNOT3*, *CNOT4* and *CNOT6* led to decreased 5-ethynyl-2′-deoxyuridine (EdU) incorporation in day 25 hiPSC-CMs and reduced CM number compared with control (Fig. 2A-D), thereby suggesting a general role of the CCR4-NOT complex in the regulation of CM proliferation.

A number of studies (Itzhaki et al., 2011; Matsa et al., 2011; Lahti et al., 2012) have shown that hiPSC-CMs from patients with long-QT syndromes consistently show prolonged APD phenotypes, suggesting that APD modulation in hiPSC-CMs represents a reliable model system to evaluate the role of candidate genes for QT-interval modulation. Therefore, we asked whether the CCR4-NOT complex could also have such a role and transfected day 25 hiPSC-CMs with siRNAs directed against each member of the complex; APD parameters were determined using a fluorescence-based, single-cell and high-throughput voltage transient recording assay, based on that used in a previous study (McKeithan et al., 2017; Fig. 2E). Interestingly, we found that *CNOT7*, but not *CNOT1*, knockdown led to a significant shortening (>20 ms) of APD (Fig. 2F-I). Although it is possible that the level of *CNOT1* knockdown was insufficient to cause a change in APD, it did produce a proliferation deficit, indicating that the siRNAs were transfected and active in hiPSC-CMs. This finding suggests a potential new role for *CNOT7* and deadenylation in the regulation of the QT interval in humans, an observation supported by the suggestive association of a variant (rs183286310; *P*=1.1×10^−6^) near the *CNOT7* gene with QT interval in ∼5000 individuals within the CHRIS study (Pattaro et al., 2015).

### Cardiac-specific *in vivo* knockdown of *CNOT1/Not1* and *CNOT7/8/Pop2* in *Drosophila* results in dilated cardiomyopathy

As experiments with hiPSC-CMs provided evidence that *CNOT1* and *CNOT7* regulate CM proliferation and APD, respectively, we considered how the same manipulations would affect the heart *in vivo*. The CNOT genes are conserved in *Drosophila* and have the following orthologs to the human CNOT genes: *CNOT2*/*Not2*, *CNOT4*/*Not4*, *CNOT6* and *CNOT6L*/*twin*, *CNOT7* and *CNOT8*/*Pop2* (Table 1).

**Table 1.**
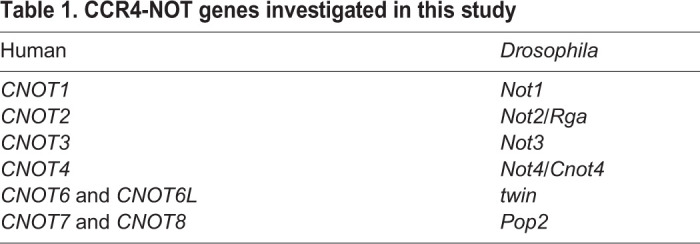
**CCR4-NOT genes investigated in this study**

**Fig. 3.**
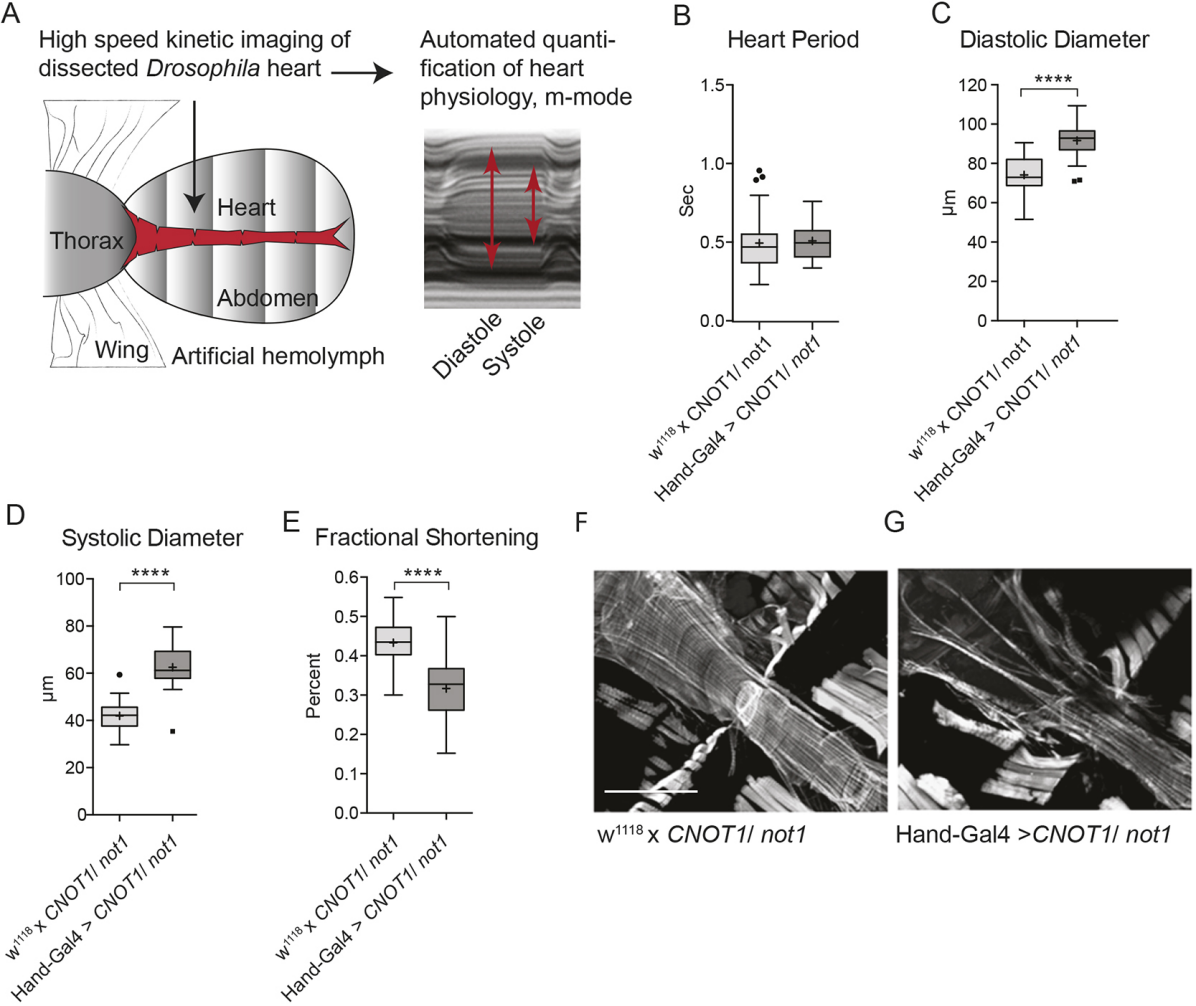
**Cardiac-specific *in vivo* knockdown of *CNOT1/Not1* in *Drosophila*.** (A) Schematic of heart analysis procedure in *Drosophila*. Dissection in artificial hemolymph exposes the beating fly heart for video recording. Automated quantification generates M-modes used to measure heart period, heart size and contractility. (B-E) RNAi-mediated knockdown of *CNOT1*/*Not1* (VDRC, GD12571) using the cardiomyocyte and pericardial cell-specific driver *Hand-Gal4*; *n*=40 female flies per genotype. Reduced *CNOT1*/*Not1* expression increased diastolic and systolic diameters and reduced fractional shortening. Student's *t*-test was used to calculate two-sided *P*-values. ****P≤0.0001. Boxes, interquartile range; central line, median; plus, mean; whiskers, upper and lower adjacent values as defined by Tukey (1977); dots, outside values. (F,G) Immunofluorescence staining with phalloidin visualized F-actin of *Drosophila* hearts. A representative *Not1* knockdown heart shows myofibrillar disarray and gaps in muscle tissue compared with the tightly packed circumferential myofibrils seen in the control heart. Scale bar: 200 µm.

Using the *Drosophila* UAS-Gal4 system (Brand and Perrimon, 1993) we silenced *CNOT1*/*Not1* with *Hand-Gal4*, a driver specific for myocardial and pericardial cells of the heart that acts throughout development (Han and Olson, 2005). At 1 week post-eclosion (young adult flies), we dissected the animals to expose the heart for video recording as previously described (Ocorr et al., 2007a, 2014; Fink et al., 2009) (Fig. 3A). The *Hand-Gal4*-driven *CNOT1/Not1* knockdown hearts exhibited normal beating frequency (shown as heart period, Fig. 3B). However, these hearts were fragile and exhibited diastolic and systolic diameters that were significantly larger than controls (Fig. 3C,D). These changes resulted in a substantial decrease in fractional shortening (Fig. 3E) and a reduced capacity for the heart to contract. Fluorescent staining of actin revealed an abnormal myofibrillar structure with large gaps and disarray in *CNOT1/Not1* knockdown fly hearts compared with control hearts, displaying typical tightly packed circumferential myofibrils (Fig. 3F,G). Knockdown with two different *CNOT1/Not1* RNAi lines (GD12571 and KK106587) resulted in similar phenotypes, whereas knockdown with a third line (TRiP 28681) resulted in the same trend, but was not statistically significant from control fly hearts. The *CNOT1/Not1* RNAi line VDRC GD12571 is shown in all figures.

We had previously observed double-beat early afterdepolarization (EAD)-associated arrhythmias in M-mode traces from movies of *CNOT3/Not3* knockdown fly hearts (Neely et al., 2010), thus an effort was made to record electrophysiological traces. This proved nearly impossible, however, owing to the fragility of the *CNOT1/Not1* knockdown hearts. One successful recording did show abnormal fibrillatory events, with increased event duration and number of peaks per burst (Fig. S2A,B).

**Fig. 4.**
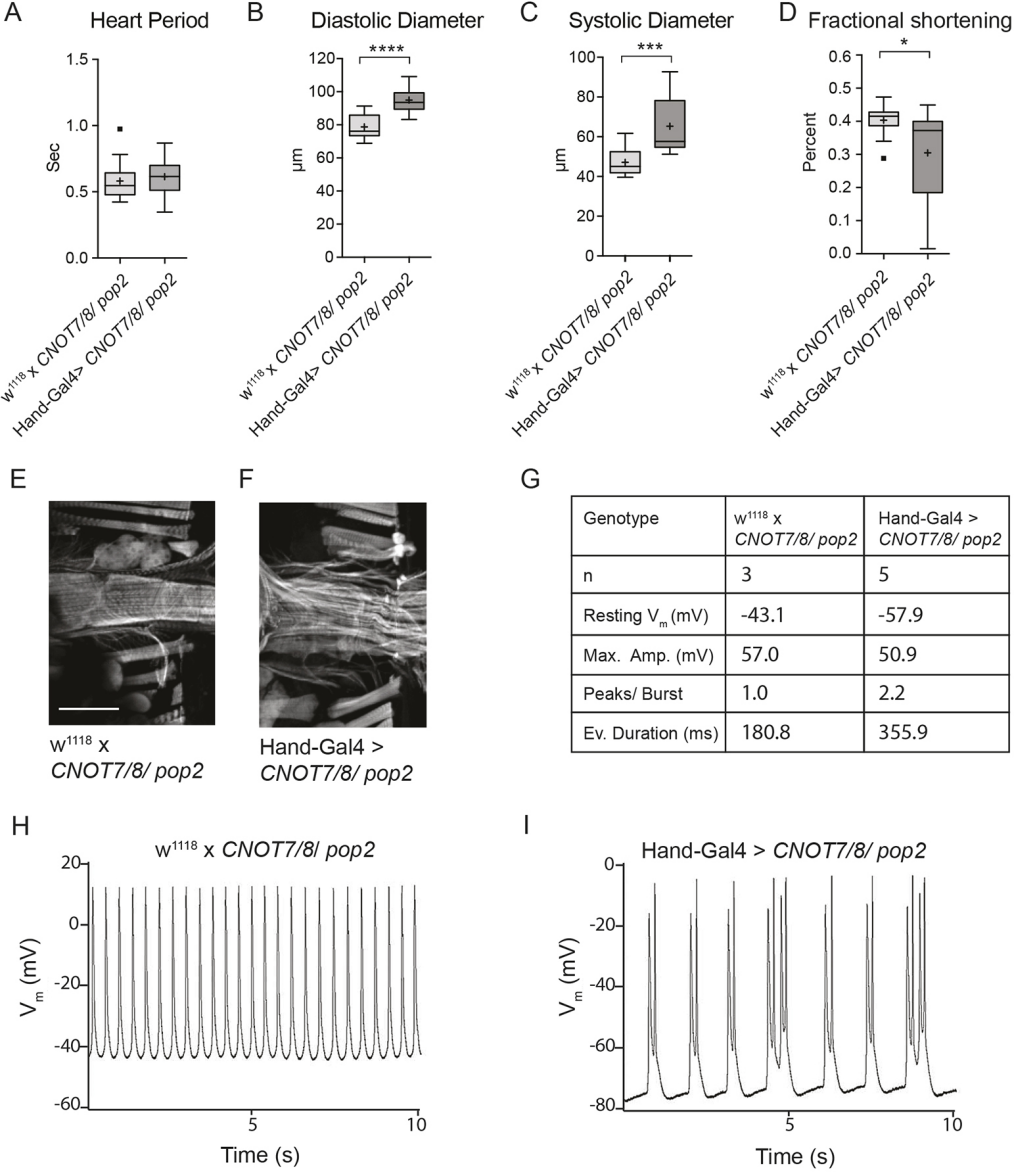
**Cardiac-specific *in vivo* knockdown of *CNOT7/8/Pop2* in *Drosophila*.** (A-D) RNAi-mediated knockdown of *CNOT7/8/Pop2* (TRiP HM05235) using the cardiomyocyte and pericardial cell specific driver *Hand-Gal4* (*n*=32 female flies per genotype). Reduced *Pop2* expression increased diastolic and systolic diameters and reduced fractional shortening. Student's *t*-test was used to calculate two-sided *P*-values. *P≤0.05, ***P≤0.001, ****P≤0.0001. Boxes, interquartile range; central line, median; plus, mean; whiskers, upper and lower adjacent values as defined by Tukey (1977); dots, outside values. (E,F) Immunofluorescence staining with phalloidin visualizes F-actin of *Drosophila* hearts. The *CNOT7/8/Pop2* knockdown heart shows dilation and gaps in muscle tissue compared with the tightly packed circumferential myofibrils in control heart. Scale bar: 100 µm. (G) Table summarizing electrophysiological measurements of *CNOT7/8/Pop2* knockdown fly hearts. (H,I) Representative 10 s M-modes show greater peaks per burst and longer event duration of *CNOT7/8*/*Pop2* knockdown (*Hand-Gal4*>*CNOT7/8*/*Pop2*) fly hearts compared with control (w^1118^×*CNOT7/8/Pop2*).

As the results of the hiPSC-CMs indicated the importance of the deadenylase *CNOT7* for cardiac rhythm control, we asked the question how knockdown of the *Drosophila* ortholog *CNOT7/8/Pop2* would affect the fly heart. RNAi-mediated knockdown of *CNOT7/8/Pop2*, through the *Hand-Gal4* driver line, resulted in pupal lethality at 25°C. By lowering the incubation temperature during development to 18°C, and thereby reducing Gal4 production, the flies did eclose. When 1-week-old *CNOT7/8/Pop2* knockdown hearts were functionally analyzed, they exhibited no change in heart period (Fig. 4A). However, significant cardiac dilation, as measured by increased diastolic and systolic diameters (Fig. 4B,C), and reduced contractility (Fig. 4D) were evident, consistent with the phenotype observed in *CNOT1*/*Not1* knockdown fly hearts. Furthermore, fluorescent staining of *CNOT7/8/Pop2* knockdown hearts revealed myofibrillar structural abnormalities (Fig. 4E,F), as seen in *CNOT1/Not1* knockdown hearts (Fig. 3F,G). Electrophysiological recordings indicated that cardiac-restricted silencing of *CNOT7/Pop2*, as observed with the single *CNOT1/Not1* knockdown heart, triggered longer event durations and multiple peaks per burst (Fig. 4G-I) compared with control hearts. Although we cannot be certain that the single *CNOT1/Not1* recording is representative, taken together with the *CNOT7/8/Pop2* electrophysiological recordings, we find that this phenotype is consistent with a propensity for arrhythmia.

**Fig. 5.**
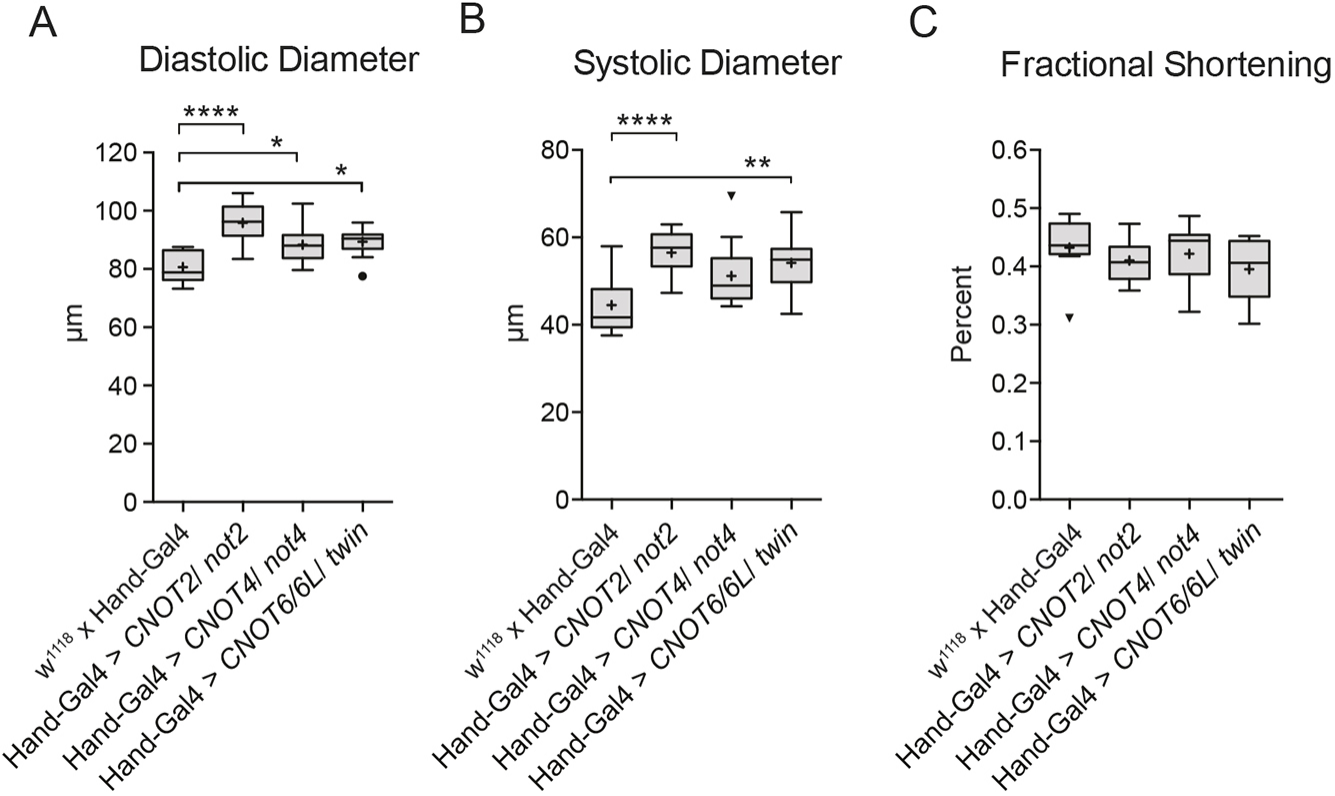
**RNAi-mediated knockdown of *CNOT2/Not2* (VDRC GD20826), *CNOT4/Not4* (TRiP JF03203) and *CNOT6/6L/twin* (VDRC GD13365) using the cardiomyocyte and pericardial cell specific driver *Hand-Gal4***. (A) Reduced expression of *CNOT2/Not2*, *CNOT4/Not4* and *CNOT6/6L/twin* significantly increased diastolic diameter. (B) Reduced expression of *CNOT2/Not2* and *CNOT6/6L/twin* significantly increased systolic diameter. (C) Fractional shortening was not changed by reduced expression of either *CNOT2/Not2*, *CNOT4/Not4* or *CNOT6/6L/twin.* *n*=15 female flies per genotype. One-way ANOVA with Tukey's multiple comparisons test was used to calculate two-sided *P*-values. *P≤0.05, **P≤0.01, ****P≤0.0001. Boxes, interquartile range; central line, median; plus, mean; whiskers, upper and lower adjacent values as defined by Tukey (1977); dots/arrowheads, outside values.

*Hand-Gal4*-driven RNAi knockdown of *CNOT2/Not2*, *CNOT4/Not4* and *CNOT6/6L/twin* also caused cardiac dilation, as observed with the silencing of *CNOT1/Not1* and *CNOT7/8/Pop2*. Knockdown of *CNOT2/Not2*, *CNOT4/Not4* and *CNOT6/6L/twin* induced a significant increase in diastolic diameter (Fig. 5A), but only *CNOT2/Not2* and *CNOT6/6L/twin* silencing also resulted in increased systolic diameters compared with controls (Fig. 5B). This dilation did not result in significantly diminished fractional shortening, however (Fig. 5C). The results suggest that *CNOT2/Not2*, *CNOT4/Not4* and *CNOT6/6L/twin* are needed for normal heart dimensions *in vivo*, but their reduction did not significantly affect overall contractility.

We repeated all RNAi experiments with a second driver line, *TinCΔ4-Gal4* (Lo and Frasch, 2001), which is expressed in the myocardium during early development, during late pupal stages of cardiac remodeling and in the adult heart, but not during larval and early pupal stages. Surprisingly, *TinCΔ4-Gal4*-mediated *CNOT1/Not1* knockdown did not cause a dilated cardiac phenotype, except for a small increase in systolic heart diameter that resulted in modestly decreased fractional shortening (Fig. S2C-E). *TinCΔ4-Gal4-*driven knockdown of *CNOT2/Not2*, *CNOT4/Not4* and *CNOT6/6L/twin* did not engender a cardiac phenotype compared with controls, although *CNO4/Not4* knockdown resulted in substantially increased diastolic and systolic diameters, but with no effect on fractional shortening (Fig. S3).

### Knockdown of *CNOT1/Not1* and *CNOT7/8/Pop2* during *Drosophila* development (larval stages)

**Fig. 6.**
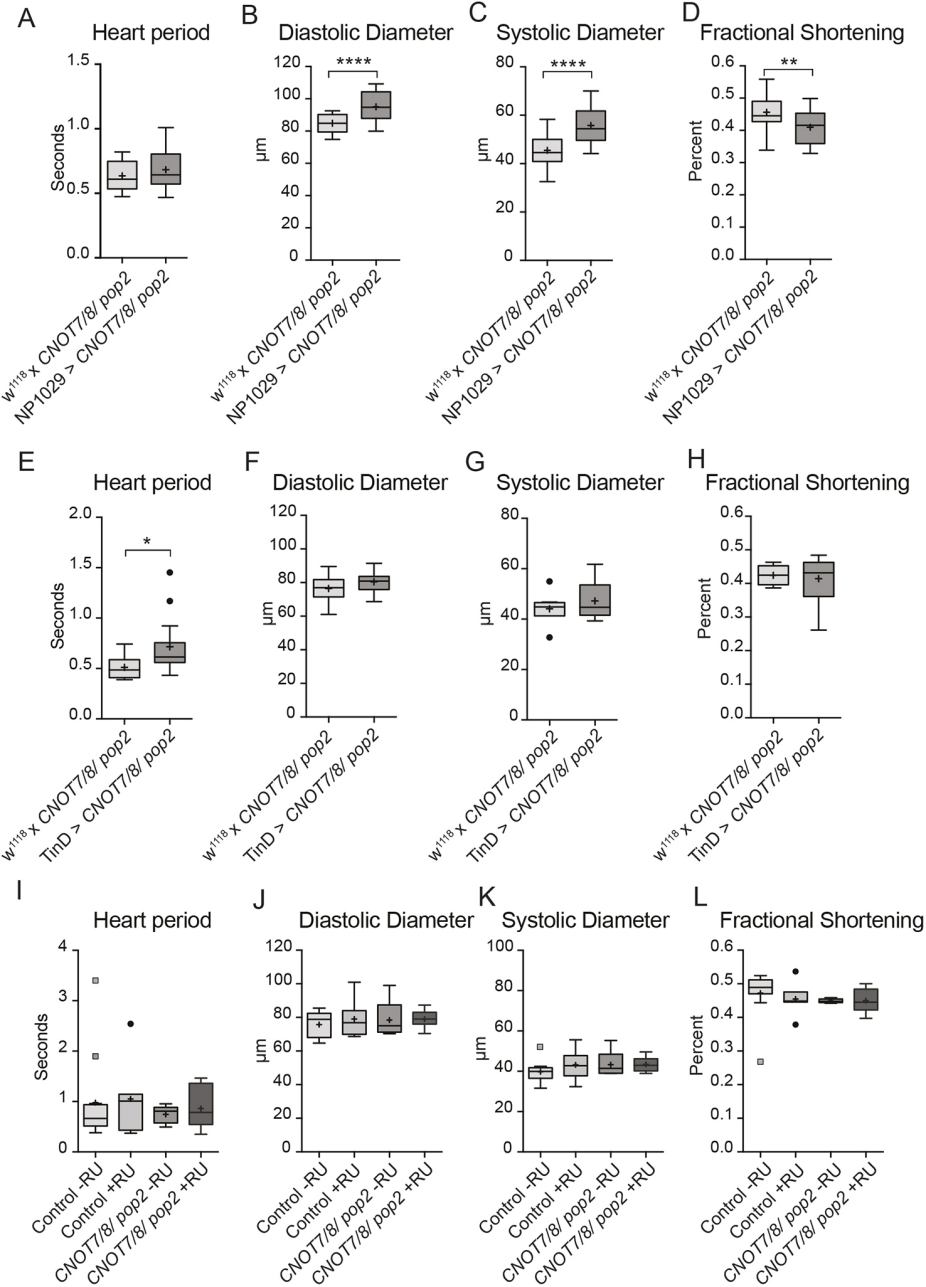
**RNAi-mediated knockdown of *CNOT7/8/Pop2* (TRiP HM05235) during developmental stages and in adult fly hearts.** (A-D) RNAi-mediated knockdown of *CNOT7/8/Pop2* using the larval stage heart-specific driver *NP1029-Gal4* (*n*=19 female flies per genotype). Reduced *CNOT7/8/Pop2* expression increased diastolic and systolic diameters and reduced fractional shortening. (E-H) RNAi-mediated knockdown of *CNOT7/8/Pop2* using embryonal stage heart-specific driver *TinD-Gal4* extended heart period, but had no effect on heart diameters and contractility (*n*=19 female flies per genotype). Student's *t*-test was used to calculate two-sided *P*-values in A-D and E-H. *P≤0.05, **P≤0.01, ****P≤0.0001. (I-L) RNAi-mediated knockdown of *CNOT7/8/Pop2* using the RU-486 inducible *Hand-Gal4* Gene Switch driver. Reduced expression of *CNOT7/8/Pop2* in adult fly hearts had no effect on heart diameters or contractility (*n*=12 female flies per condition and genotype). Two-way ANOVA was used to calculate *P*-values. Boxes, interquartile range; central line, median; plus, mean; whiskers, upper and lower adjacent values as defined by Tukey (1977); dots, outside values.

The finding that knockdown of *CNOT1/Not1* and *CNOT7/8/Pop2* with the pupal/adult *TinCΔ4-Gal4* myocardial driver (not expressed in larval hearts) did not recapitulate the results obtained with the continuously expressed *Hand-Gal4* heart driver raised the question as to whether developmental expression in larvae/early pupae was crucial for normal adult heart function. We therefore tested the hypothesis that the discrepancies observed between the drivers were due to temporal expression differences. To test our hypothesis, we used the driver *NP1029-Gal4* that conferred larval/early pupal-specific gene silencing (Monier et al., 2005). *CNOT1/Not1* knockdown using *NP1019-Gal4* was partially larval lethal and completely pupal lethal at 25°C, whereas *CNOT7/8/Pop2* knockdown flies did eclose. Analysis of 1-week-old fly hearts upon larval/early pupal *CNOT7/8/Pop2* knockdown revealed significant dilation and reduction in fractional shortening and normal heart period (Fig. 6A-D), similar to the results obtained with the *Hand-Gal4* driver. Although we did not test for a cardiac phenotype at early pupal stages, it is unlikely to be manifest similarly at adult stages, because most of the larval heart will undergo histolysis and more anteriorly located portions of the larval aorta will metamorphose during later pupal stages to become the adult heart (see Monier et al., 2005). Thus, the observed adult heart phenotype is expected to be established during later pupal stages, although *CNOT7/8/Pop2* function is already required during larval/early pupal stages and perhaps even in the embryo.

To test this idea, we further explored the developmental requirements by knocking down *CNOT7/8/Pop2* in the cardiac mesoderm during early embryonal stages using the driver line *Tin-D-Gal4* (Reim and Frasch, 2005). This knockdown did not have any significant effects, except for a prolonged heart period in *CNOT7/8/Pop2* knockdown flies compared with control (Fig. 6E-H). The converse experiment was also performed, which was to knockdown *CNOT7/8/Pop2* in adult flies with the *Hand-Gal4* Gene Switch system (Monnier et al., 2012), which activates RNAi-mediated silencing in the myocardial and pericardial cells only when induced with RU-486. Adult flies were placed in food vials containing RU-486 at eclosion and were analyzed at 1 week of age. Knockdown of *CNOT7/8/Pop2* in adult flies had no statistically significant effect on any of the cardiac parameters measured (Fig. 6I-L).

Taken together, our data strongly suggest that the CCR4-NOT complex, in particular *CNOT1/Not1* and *CNOT7/8/Pop2*, along with *CNOT3* (Neely et al., 2010; Yamaguchi et al., 2018), are required during the larval and/or early pupal stages of development, and thus during the initial stages of cardiac remodeling from the larval to adult heart.

## DISCUSSION

GWAS studies have successfully identified many genetic loci associated with multiple disorders, including cardiovascular disease (Buniello et al., 2019). Nevertheless, how to use GWAS results to recognize the specific targets of these associations and for understanding the biology of disease remains a major challenge. Progress in this area will enable the future improvement of diagnostics and personalized therapy. Here, we started with QT interval-associating variants in the *CNOT1* gene, an integral component of the CCR4-NOT complex, and expanded on this observation to include an investigation of additional complex subunits. A combined approach using hiPSC-CMs and *Drosophila* enabled a human and whole-organ assessment of cardiac physiology.

We examined the functionality of human variants associated with QT interval in the *CNOT1* promoter region and determined that the alleles of variants that significantly associate with increases in QT interval are capable of lowering reporter gene expression, which might also reflect reduced transcription of *CNOT1*. We do note, however, that the human SNPs tested *in vitro* are located in the *CNOT1* promoter region, and that mutation of the actual gene might have different consequences in humans as compared with altered gene expression in cardiac tissue alone. As *CNOT1* is essential for the function of the CCR4-NOT complex (Ito et al., 2011), we expanded our study to include other subunits to assess the range of potential functional differences. We found that knockdown of not only *CNOT1*, but also *CNOT2*, *CNOT3*, *CNOT6*, *CNOT6L* and *CNOT7*, decreased proliferation of hiPSC-CMs, and that knockdown of *CNOT7* also caused significant APD shortening. Knockdown of *CNOT8* did not affect CM proliferation, which might be due to compensation by CNOT7 (Fig. 2).

Consistent with our observations in hiSPCs, *in vivo* findings show that *CNOT1/Not1* knockdown flies exhibit dilated hearts with reduced contractile ability and severe structural defects, similar to the myofibrillar reduction and cardiomyocyte death observed in *CNOT1* and *CNOT3* muscle-specific knockout mice (Yamaguchi et al., 2018). Importantly, silencing of *CNOT7/8/Pop2* resulted in cardiac damage similar to *CNOT1/Not1* knockdown, and electrophysiological recordings demonstrated extended event duration and multiple peaks per burst, which is indicative of a propensity for arrhythmias. Muscle tissue defects and electrical activity have been linked in mouse and humans (Chinchilla and Franco, 2006). Moreover, mutations in *seizure* [the *Drosophila* homolog of the human K^+^ channel gene *hERG* (also known as *KCNH2*), which is important in cardiac repolarization] not only cause bradycardia and arrhythmia, but also structural defects such as myofibrillar disorganization (Ocorr et al., 2017). Silencing of *CNOT2/Not2*, *CNOT4/Not4* and *CNOT6/6L/twin* subunits led overall to similar, albeit weaker, phenotypes limited to increased diastolic and systolic diameters. Importantly, the role of CNOT subunits in the action potential repolarization phase was demonstrated not only in the *Drosophila* heart model (Ocorr et al., 2017), but also in humans (Tse et al., 2017).

Notably, in our hands, dilated cardiomyopathy (DCM) resulted as the main phenotype produced by *Hand-Gal4-*driven knockdown of *CNOT/Not1* in the *Drosophila* heart. Unfortunately, transthoracic echocardiography, the first-line imaging test in the assessment of ventricular dilation (Mathew et al., 2017), was not performed in the individuals from whom we isolated the natural variants of the *CNOT1* promoter; thus, we could not evaluate structural alterations in those individuals. However, genetic-based forms of long-QT have been associated with the development of DCM. An overlap between DCM and long-QT3 resulting from abnormalities of the sodium channel gene *SCN5A* have been described in multiple reports (Kwon et al., 2012; Shi et al., 2008), but there is also evidence for an association between long-QT1 and idiopathic DCM (Allen et al., 2016). In addition, it has been reported that patients with both severe or mild forms of cardiomyopathies, such as DCM or hypertrophic cardiomyopathy, can show QT prolongation (Johnson et al., 2011; Jouven et al., 2002; Ryerson and Giuffre, 2006). Of note, the role of the causative mutation in the overlap between channelopathies and cardiomyopathies is not fully understood, and the role of possible new players acting as phenotype modifiers, like the CCR4-NOT complex, has yet to be determined.

When using a cardiac driver for gene knockdown that excluded the larval and early pupal stages of fly heart development (*TinCd4-Gal4*), we failed to observe a strong requirement for *CNOT1/Not1* and *CNOT7/8/Pop2*. In contrast, when using a driver that was restricted specifically to larval and early pupal stages (*NP1029-Gal4*), *CNOT1/Not1* knockdown was lethal; *CNOT7/8/Pop2* silencing at this stage of development resulted in dilation and reduced contractility, similar to knockdown exerted throughout life. An embryonic or adult-only driver had no effect on cardiac outcome for either gene. These findings suggest that CCR4-NOT function is crucial during cardiac remodeling from the larval to the adult heart. It is, however, also possible that knockdown in adult flies would have an effect under stress conditions.

Considering the effects on hiPSC-CM proliferation and the developmental defects observed by silencing *CNOT1/Not1* and *CNOT7/8/Pop2* in *Drosophila*, we speculate that the CCR4-NOT-encoding genetic variants identified by GWAS in adult humans are those that result in less severe consequences, as the lack of proper mRNA regulation might be lethal at certain stages of embryogenesis. Depletion of *CNOT1* and the entire deadenylase module (*CNOT6/6L*, *CNOT7/8*), has been demonstrated to promote endoplasmic reticulum (ER) stress and apoptosis *in vitro* (Ito et al., 2011). In turn, it has also been shown that activation of the unfolded protein response impairs cardiac ion channel biogenesis, leading to a prolongation of the APD (Liu et al., 2018). Taken together, these findings suggest that disruption of CCR4-NOT complex function affects both structural (i.e. decreased CM proliferation, myofibrillar structural abnormalities) and electrophysiological (i.e. shortened APD, decreased contractility in flies) components of the heart. Whether knockdown of a specific subunit produces one or both of these phenotypes might be influenced by silencing efficiency; for example, less ER stress might lead to electrical remodeling, whereas more might result in apoptosis. In addition, specific RNA-binding proteins that connect and guide CCR4-NOT to target-specific mRNAs are likely to have an influence. Collectively, our results show a prominent role of the deadenylase module (*CNOT7/8/Pop2*) both *in vitro* and *in vivo.*

The combined use of GWAS studies and cardiac model systems in this study has enabled us to connect *CNOT1*, *CNOT7* and, overall, CCR4-NOT complex function to cellular and whole-heart phenotypes in the context of human heart disease. In this context, however, direct CCR4-NOT complex targets that influence heart rhythm and physiology remain to be identified. Finally, strategies to modulate the expression of key components of the CCR4-NOT complex, or to stabilize its function, might be promising avenues for regulating QT interval and preventing pro-arrhythmogenic substrates, especially targeted to those individuals at increased risk owing to their genetic background.

## MATERIALS AND METHODS

### Ethics statement

The DNA for promoter isolation was drawn from individual participants in the MICROS study in South Tyrol (Pattaro et al., 2007). MICROS was approved by the Ethics Committee of the Autonomous Province of Bolzano (Südtiroler Sanitätsbetrieb/Azienda Sanitaria dell'Alto Adige). Each participant gave written informed consent.

### Generation of hiPSC-CMs

hiPSC-CMs were dissociated with 0.5 mM EDTA in PBS without CaCl_2_ and MgCl_2_ (Corning) for 7 min at room temperature (RT), resuspended in mTeSR-1 medium (StemCell Technologies) with 2 µM thiazovivin (StemCell Technologies) and 3×10^5^ cells/well were plated in a Matrigel-coated 12-well plate. At 24 h after passage, cells were fed daily with mTeSR-1 medium (without thiazovivin) for 3-5 days until ≥90% confluence. hiPSC-CMs were differentiated as previously described (Burridge et al., 2015). On day 0, WNT signaling was activated by adding 6 µM CHIR99021 (Selleck Chemicals) in S12 medium (Pei et al., 2017) for 48 h. On day 2, cells were treated with 2 µM Wnt-C59 (Selleck Chemicals) in S12 to inhibit WNT. On day 4, S12 medium was fully changed. On day 5, cells were dissociated with TrypLE Express (Gibco) for 4 min and blocked with RPMI (Gibco)+10% fetal bovine serum (FBS; Omega Scientific). Cells were resuspended in S12 supplemented with 4 mg/l recombinant human insulin (Gibco) (S12+ medium) and 2 µM thiazovivin and 9×10^5^ cells/well were plated in a Matrigel-coated 12-well plate. S12+ medium was changed on day 8 and replaced on day 10 by RPMI (Gibco)+213 µg/µl L-ascorbic acid (Sigma-Aldrich), 500 mg/l BSA-FV (Gibco), 0.5 mM L-carnitine (Sigma-Aldrich) and 8 g/l AlbuMAX lipid-rich BSA (Gibco) (CM medium). Under these conditions, hiPSC-CMs start to beat around day 9-10. On day 15, cells were purified with lactate medium, consisting of RPMI without glucose, 213 µg/µl L-ascorbic acid, 500 mg/l BSA-FV and 8 mM sodium-DL-lactate (Sigma-Aldrich) (Burridge et al., 2015; Tohyama et al., 2016), for 4-5 days and was replaced by CM medium until day 25.

### Proliferation assay in hiPSC-CMs

At day 25 of differentiation, hiPSC-CMs were dissociated with TrypLE Select 10× (Gibco) for 12 min and neutralized with RPMI+10% FBS. Cells were resuspended in RPMI with 2% KnockOut Serum Replacement (KOSR; Gibco) and 2% B27 50× with vitamin A (Life Technologies) supplemented with 2 µM thiazovivin and plated at a density of 5000 cells/well in a Matrigel-coated 384-well plate. hiPSC-CMs were transfected with siRNA (Dharmacon) targeting siCNOT1 (L-015369-01), siCNOT2 (L020313-02), siCNOT3 (L-020319-00), siCNOT4 (L-020323-00), siNOT6 (L-019101-00), siNOT6L (L-016411-00), siCNOT7 (L-012897-00) and siCNOT8 (L-018791-00), using lipofectamine RNAiMax (Thermo Fisher Scientific). Each siRNA was tested in quadruplicate. Cells were labeled with 10 µM EdU (Thermo Fisher Scientific) 48 h post-transfection. After 24 h of EdU incubation, cells were fixed with 4% paraformaldehyde for 30 min. EdU was detected according to protocol and cells were stained with the cardiac-specific marker ACTN2 (Sigma-Aldrich, dilution 1/800) and DAPI. Cells were imaged with an ImageXpress Micro XLS microscope (Molecular Devices) and custom algorithms were used to quantify EdU-labeled hiPSC-CMs.

### Voltage assay in hiPSC-CMs

Voltage assay was performed as described in McKeithan et al. (2017). At day 25 of differentiation, hiPSC-CMs were dissociated with TrypLE Select 10× for up to 12 min and neutralized with RPMI+10% FBS. Cells were resuspended in RPMI with 2% KOSR (Gibco), 2% B27 50× with vitamin A (Life Technologies) and supplemented with 2 µM thiazovivin and plated at 6000 cells/well in a Matrigel-coated 384-well plate. hiPSC-CMs were transfected with CCR4-NOT-NOT siRNAs as described above. Three days post-transfection, cells were washed five times with pre-warmed Tyrode's solution (Sigma-Aldrich) by removing 50 µl of medium and adding 50 µl. After the fifth wash, 50 µl of 2× dye solution [voltage-sensitive dye Vf2.1 Cl (Fluovolt, 1:4000, Thermo Fisher Scientific) diluted in Tyrode's solution with 1 µl of 10% pluronic F127 (in water, Thermo Fisher Scientific) and 20 µg/ml Hoechst 33258 (in water, Thermo Fisher Scientific)] was added to each well. The plate was returned to an incubator at 37°C 5% CO_2_ for 45 min. After incubation, cells were washed four times with pre-warmed Tyrode's solution. hiPSC-CMs were imaged with an ImageXpress Micro XLS microscope at 100 Hz for 5 s, with excitation wavelength at 485/20 nm and emission filter 525/30 nm. A single image of Hoechst was acquired before the time series. Fluorescence over time quantification and trace analysis were automatically quantified using custom software packages developed by Molecular Devices and the Colas Laboratory. Three independent experiments were performed, each condition in quadruplicate.

### Cell culture

HL-1 mouse atrial cardiomyocytes (Claycomb et al., 1998) were kindly donated by William Claycomb (Louisiana State University, New Orleans, LA, USA) and cultured in Claycomb medium (Sigma-Aldrich) supplemented with 10% FBS, 4 mM L-glutamine, 100 U/ml penicillin, 100 mg/ml streptomycin, 0.3 mM ascorbic acid and 10 mM norepinephrine, as previously described (Meraviglia et al., 2015).

HeLa and 293T cells were cultured in Dulbecco's modified Eagle medium (DMEM), GlutaMAX supplement (Thermo Fisher Scientific), supplemented with 10% FBS (Sigma-Aldrich) and 1% penicillin-streptomycin (Thermo Fisher Scientific). All cells were maintained at 37°C in a saturated humidity atmosphere containing 5% CO_2_.

### Luciferase assay

HL1, HeLa and T293 cells were seeded at 30,000, 60,000 and 150,000 cells/well, respectively, into 24-well plates (Corning). A total of 24 h after seeding, 10 ng of the reporter plasmid pG4.74[hRluc/TK] was co-transfected with 10 ng of either pGL4.10 vector (complete or minimal for both haplotypes) or negative control vector (pG4.13 [luc2/SV40]). Transfection was performed with the lipofectamine plus reagent (Invitrogen), according to the manufacturer's protocol. Cells were washed with PBS 48 h post-transfection and lysed with 100 μl of passive lysis buffer (Promega) for 15 min at RT. Cell lysates were immediately used to measure luciferase activity, using the Dual Luciferase Reporter Assay System Kit (Promega). Each lysate (20 μl) was incubated with 100 μl of luciferase assay reagent II (LAR II). Firefly luminescence was measured for 10 s using a luminometer (Victor X3-2030, Perkin Elmer). After 2 s, 100 μl of Dual-Glo Stop and Glo Reagent was added to each well. Subsequently, renilla luminescence was measured for 10 s using the same luminometer. Luciferase activity was calculated based on the ratio of the activities of firefly and renilla luciferases. At least three independent experiments were performed in triplicate.

### Fly stocks

All transgenic RNAi fly lines were purchased from Vienna *Drosophila* RNAi Center (VDRC) and from Bloomington Drosophila Stock Center (BDSC) at Indiana University (Transgenic RNAi Project at Harvard Medical School, TRiP). VDRC IDs: *Not1* (GD12571 and KK106587), *Not2* (GD20826), *Pop2* (GD28396) and *twin* (GD13365). TRiP/BDSC IDs: *Not1* (28681), *Not4* (JF03203), *Pop2* (HM05235) and *twin* (HMS00493). Control flies with corresponding genetic background: VDRC *w^1118^* (GD RNAi library) and TRiP-fly line with attP2 docking site. Cardiac-specific drivers were kind gifts from the following investigators: Manfred Frasch, *TinCΔ4 12a-Gal4* (Lo and Frasch, 2001) and *tin-D-Gal4* (Reim and Frasch, 2005); Eric Olsen, *Hand-Gal4* (Han and Olson, 2005); Laurent Perrin, *NP1029-Gal4* (Monier et al., 2005) and *Hand-Gal4 Gene Switch* (Monnier et al., 2012).

### Fly medium

Ingredients of fly medium comprised cornmeal (7.0%), malt (5.2%), molasses (5.2%), soy flour (1.7%), agar (0.4%) and autolyzed yeast (2.1%). All ingredients were mixed with water and cooked for 15 min without boiling. Preservatives, Tegosept in ethanol (1.8%) and propionic acid (2.1%) were added once the batter cooled to <65°C. All percentages refer to the final concentration. Dry ingredients, weight/total volume batter; liquid ingredients, volume/total volume batter.

### Fly crosses

Driver-line virgins were crossed to RNAi males and corresponding isogenic control males. Flies were raised on standard fly food and kept at 25°C or 18°C. Female progeny was collected and aged to 1 week at 25°C, at which point they were imaged and analyzed.

### *Hand-Gal4* gene-switch knockdown

Eclosed female progeny were collected and placed in vials with fly food containing 100 µg/ml RU-486 (40 µl of 25 mg/ml stock RU-486 dissolved in ethanol was added to 10 ml fly food). Control food contained an equal amount of ethanol. Flies were aged to 1 week at 25°C, at which point they were dissected and analyzed.

### Fly heart dissection

Flies were anesthetized with FlyNap and dissected in artificial hemolymph according to a previously described protocol (Vogler and Ocorr, 2009). The procedure includes removing the fly head, intestines and some fat, resulting in a semi-intact preparation that visualizes the beating heart. Artificial hemolymph was re-oxygenated for 20 min post-dissection allowing the hearts to stabilize before video recording.

### High-speed digital video imaging and analysis

All fly hearts were filmed with an EM-CDD Hamamatsu digital camera, using a Leica DMFLSA microscope equipped with a 10× dipping lens. Recordings of each fly heart were made for 30 s with a camera speed of 120-140 frames per second (Ocorr et al., 2007b). M-modes describing fly heart contractions were created by semi-automated optical heartbeat analysis (SOHA; Fink et al., 2009).

### Fluorescence staining and imaging

According to a previously described protocol (Alayari et al., 2009), flies were dissected in artificial hemolymph and hearts relaxed with 10 mM EGTA before fixation with formaldehyde. Flies were stained with phalloidin, Alexa Fluor 488 to visualize F-actin. Apotome images were taken with a Zeiss Axio Imager.Z1 microscope at 10× and 25× magnification. Images were processed with Adobe Photoshop.

### Statistical analysis

To determine any statistical significance between experimental and control groups in hiPSC-CMs and *Drosophila* experiments, we calculated two-sided *P*-values with Student's *t*-test, or one-way or two-way ANOVA with Tukey's multiple comparisons test, using GraphPad Prism software (2016). We analyzed *CNOT1* expression data from HL-1, HeLa and T293 cell lines with a two-sided non-parametric Wilcoxon rank-sum test, using Stata 13 (StataCorp. 2013; Stata Statistical Software Release 13; StataCorp, College Station, TX, USA). Population distribution of control and *siCNOT7*-transfected hiPSC-CMs was generated with GraphPad Prism using nonlinear regression. Unpaired nonparametric Kolmogorov–Smirnov test was used to compare each treated condition with controls using APD75 of every measured cell.

### Electrophysiology of adult hearts

Semi-intact heart preparations were incubated in artificial hemolymph containing 10 µM blebbistatin (Sigma-Aldrich) and left in the dark with oxygenation until the hearts stopped. Fresh saline without blebbistatin was added and electrical potentials were recorded from the conical chamber using glass electrodes (20-50 MΩ) filled with 3 M KCl. Data were acquired using an Axon-700B amplifier, signals were digitized using the DIGIDATA 1322A and data were captured and analyzed using PClamp 9.0 and Clampfit 10.0 software from Molecular Devices. Data were quantified from representative 30 s recordings where the resting membrane potential had remained stable.

## Supplementary Material

Supplementary information
