# Supplementary material for: Silencing of CCR4-NOT complex subunits affects heart structure and function
Source: Dis Model Mech. 2020 Jul 20;13(7):dmm044727. doi: 10.1242/dmm.044727 (PMC7390626; doi:10.1242/dmm.044727)
Supplement: Supplementary information [file dmm-13-044727-s1.pdf]

Figure S1

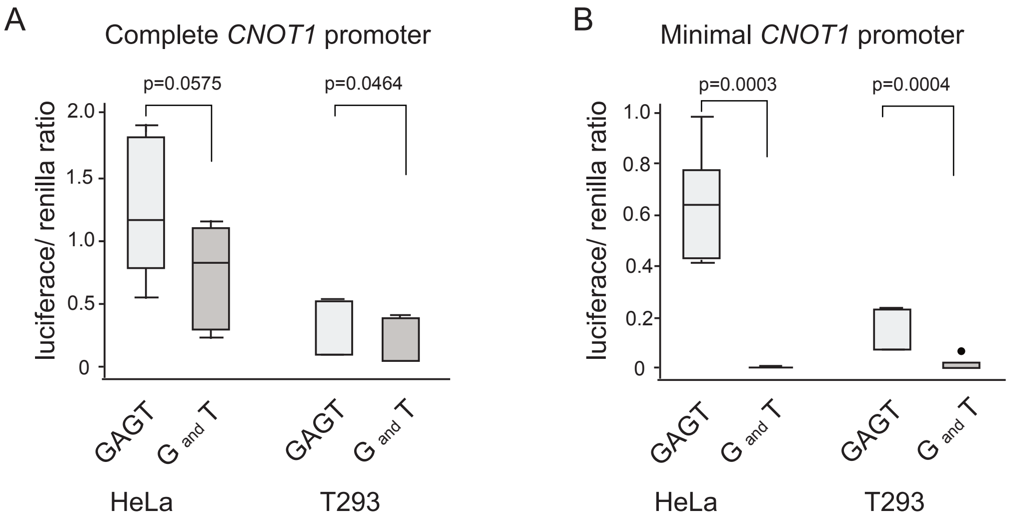

C

Marker Name	Allele1	Allele2	Effect (ms)	StdErr (ms)	P-value	Direction
rs27097	T	G	1.6218	0.2333	3.62E-12	++++++
rs37037	A	G	-0.6233	0.1997	0.001805	-+----
rs9941290	A	G	1.617	0.2313	2.71E-12	++++++
rs863433	T	G	-1.6166	0.2313	2.71E-12	------

Figure S1. Box plots summarizing the intensity of the ratio between luciferase and renilla signal in HeLa and T293 cells. **A)** Complete CNOT1 promoter construct. **B)** Minimal CNOT1 promoter construct. **C)** This table lists the four SNPs in strong linkage disequilibrium within the putative promoter region of the CNOT1 gene, with association p-values derived from the QTSCD consortium data18. This region was reported originally as the GINS3-NDRG4-SETD6-CNOT1 region as no resolution as to the functional gene in this region was possible without further experimental validation, provided herein. Allele
1 represents the effect allele identified in the GWAS meta-analysis and sequencing of this region from individuals homozygous for these variants revealed haplotypes consistent with effect direction (e.g. T at rs27097, G at rs37037, A at rs9941290 and G at rs863433) which were predicted from these results, and subsequently demonstrated experimentally, to lengthen QT interval, with the opposite haplotype predicted to shorten QT interval. The Effect column shows the observed effect in milliseconds (ms) on QT interval, and the Direction column shows the consistent direction of this effect in the six individual cohorts contributing to the QTSCD consortium.

Figure S2

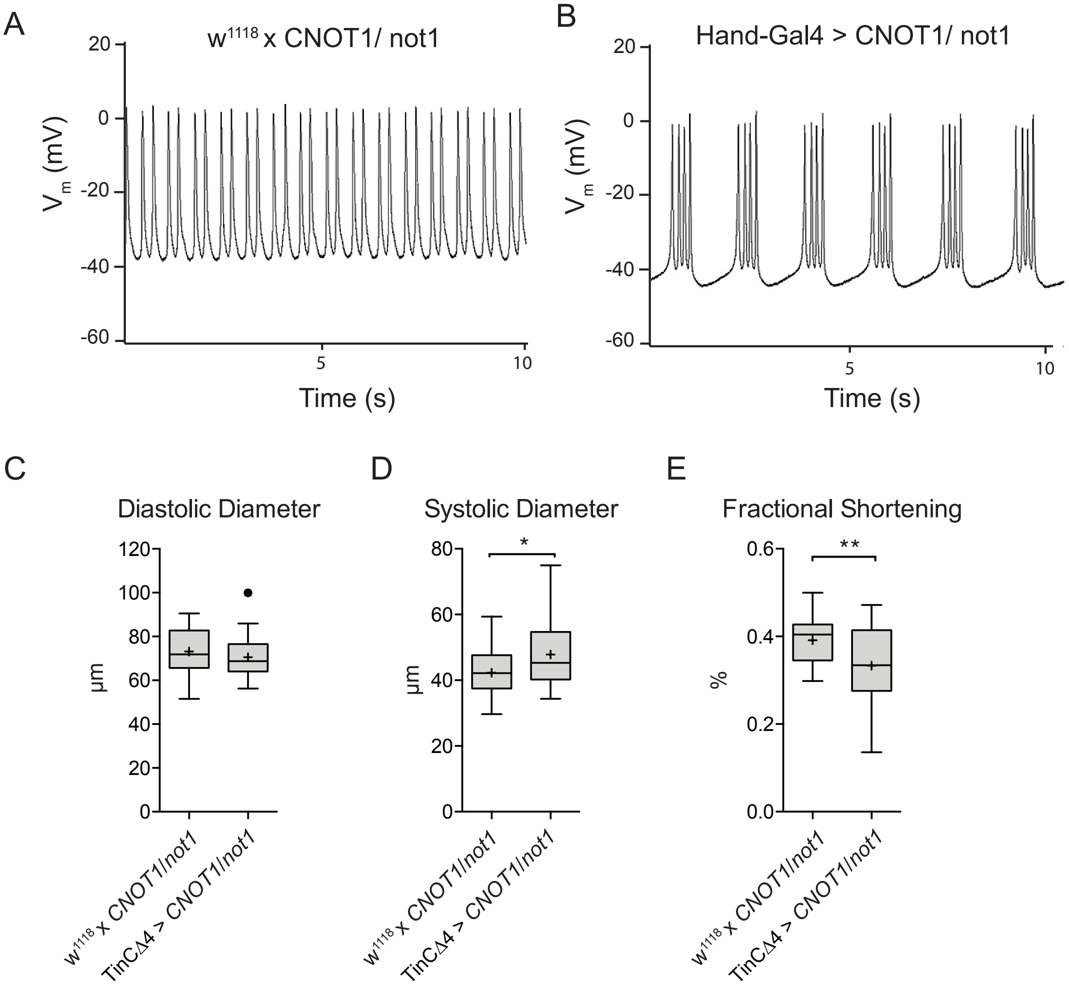

Figure S2. A-B) Electrophysiological recordings of *CNOT1/not1* (VDRC, GD12571) knockdown *Drosophila* hearts. One single electrophysiology trace obtained from fragile Hand-Gal4 driven *CNOT1/not1*-knockdown fly hearts show extended events and multiple peaks per burst com-pared to control fly. **C-E)** TinCΔ4-Gal4 driven *CNOT1/not1*-knockdown increased systolic diameter and reduced fractional shortening. n=40 female flies per genotype. Student's t-test was used to calculate two-sided p-values. Boxes: interquartile range,central line: median; plus: mean; whiskers: upper and lower adjacent values as defined by Tukey (1977); dots: outside values.

Disease Models & Mechanisms • Supplementary information

Figure S3

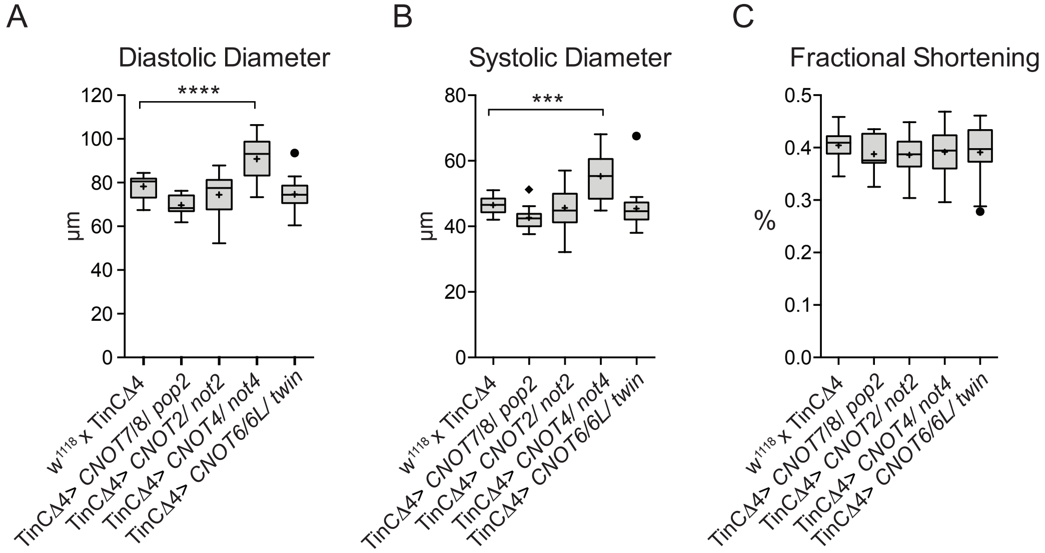

Figure S3. A-C) RNAi-mediated knockdown of *CNOT7/8/pop2* (TRiP HM05235), *CNOT2/not2* (VDRC GD20826), *CNOT4/not4* (TRiP JF03203) and *CNOT6/6L/twin* (VDRC GD13365) using cardiomyocyte specific driver TinCΔ4-Gal4 (n=15, n=20, n=19, n=20 of female flies per respective genotype). Reduced expression of *CNOT7/8/pop2*, *CNOT2/not2* and *CNOT6/6L/twin* had no effect on diastolic diameter, systolic diameter or fractional shortening. Reduced expression of *CNOT4/not4* increased both systolic and diastolic diameters but had no effect on contractility. One-way ANOVA with Tukey's multiple comparisons test was used to calculate two-sided p-values. Boxes: interquartile range, central line: median; plus: mean; whiskers: upper and lower adjacent values as defined by Tukey (1977); dots: outside values.
